# The development of a specific pathogen free (SPF) barrier colony of marmosets (*Callithrix jacchus*) for aging research

**DOI:** 10.18632/aging.101340

**Published:** 2017-12-07

**Authors:** Corinna N. Ross, Steven Austad, Kathy Brasky, Celeste J. Brown, Larry J. Forney, Jonathan A. Gelfond, Robert Lanford, Arlan Richardson, Suzette D. Tardif

**Affiliations:** ^1^ Department of Science and Mathematics, Texas A&M University San Antonio, San Antonio, TX 78224, USA; ^2^ Barshop Institute for Longevity and Aging Studies, University of Texas Health San Antonio, TX 78245, USA; ^3^ Nathan Shock Center of Excellence in the Basic Biology of Aging, Department of Biology, University of Alabama at Birmingham, Birmingham, AL 35294, USA; ^4^ Southwest National Primate Research Center, Texas Biomedical Research Institute, San Antonio, TX 78227, USA; ^5^ Department of Biological Sciences, University of Idaho, Moscow, ID 83844, USA; ^6^ Reynolds Oklahoma Center on Aging, University of Oklahoma Health Sciences Center, Oklahoma City, OK 73104, USA

**Keywords:** animal model, nonhuman primate, health span, lifespan

## Abstract

A specific pathogen free (SPF) barrier colony of breeding marmosets (Callithrix jacchus) was established at the Barshop Institute for Longevity and Aging Studies. Rodent and other animal models maintained as SPF barrier colonies have demonstrated improved health and lengthened lifespans enhancing the quality and repeatability of aging research. The marmosets were screened for two viruses and several bacterial pathogens prior to establishing the new SPF colony. Twelve founding animals successfully established a breeding colony with increased reproductive success, improved health parameters, and increased median lifespan when compared to a conventionally housed, open colony. The improved health and longevity of marmosets from the SPF barrier colony suggests that such management can be used to produce a unique resource for future studies of aging processes in a nonhuman primate model.

## INTRODUCTION

The demographics of the U.S. population is shifting such that the CDC estimates that by the year 2050 the number of people over the age of 65 will double as will the prevalence of age related disease [1]. This aging population is predicted to increase medical, economic and social burdens on society due to increased care needs. In the U.S. it is estimated that more than 60% of people over the age of 65 suffer from hypertension, roughly 40% are obese, 21% are in fair or poor health, and 7% need personal daily care [2–5]. Due to the shifting demographic needs and potential health impact on the population, research in geriatrics has recently shifted its focus from centering on human longevity to improving human health span and quality of life. Aging research is now evaluating systemic aging processes rather than concentrating on individual diseases such as diabetes, cardiovascular disease, Alzheimer's, dementia, and sarcopenia, in hopes that all of the disease progressions will be slowed with bettered health span for individuals [6, 7].

Animal models offer many advantages in studies of aging and health span research. Work with rodents has been extensive due to the fact that they are mammals that have a relatively short lifespan [8, 9]. Mice in particular have historically been used due to their ease of care, rapid developmental and reproductive rate, and the ability to create genetic mutant lines. The ease of manipulation has allowed extensive evaluation of molecular mechanisms of aging, developmental and environmental factors associated with aging, and the effects of interventional and therapeutic treatments on aging and health span in these species. The gold standard for extending life span in rodent studies is the use of caloric restriction, which has reliably increased both lifespan and health span measures in specific strains of mice [10–12]. Many new drugs of interest, including rapamycin, have been tested in rodents and continue to reveal details regarding aging and disease progression [13–15]. However, there are many concerns regarding the use of rodents as the only mammalian models of aging, since many of the drugs tested in rodents do not translate successfully to humans. Further, inbred strains of mice often have selected traits that are negative by-products of breeding [16]. Therefore, it is necessary to expand the comparative biology of aging approach in conjunction with the use of model organisms to evaluate life and health span.

Nonhuman primates offer a unique alternative for modeling questions in aging research due to their close relation to humans and the increased likelihood of translation of discoveries to human diseases. In particular, nonhuman primates may be valuable in serving as an intermediary for intervention testing, as has recently been demonstrated in the evaluation of rapamycin [17–19]. However, most nonhuman primate biomedical models have longevity longer than a researcher's career and are expensive to maintain and test.

The marmoset is a small bodied, short lived primate that has recently been developed as an alternative model for aging research [20, 21]. Prior to 2012, the average lifespan for marmosets was typically reported as approximately 5-6 y, with animals considered aged at 8 y and a maximum age of 16 y [20–22]. However, two published reports suggest that these typically reported average and maximum lifespans do not reflect the true lifespan potential of the species. Ridley, et al. [23] reported on a small breeding colony at Cambridge University in which 80% of individuals were still alive at age 10 y and the maximum lifespan was 19 y. In 2012, a marmoset colony in Japan (CLEA) reported an average lifespan of 12 y with a maximum of 22 y. The dynamics in these colonies that led to the extended lifespan is unclear, but Nishijima, et al. (2012) propose that the extended longevity in their closed colony was due to few infection related deaths.

Survival curves reported for marmosets typically exhibit a normal primate linear decline in middle age rather than the plateaued survival typical of mouse models and Western human populations [21]. Such a pattern appears typical for conventionally maintained, captive nonhuman primate populations [24]. The use of marmosets as biomedical models for aging research would be greatly enhanced if the marmoset longevity curve could be reshaped to resemble the plateaued human and mouse model curves. These plateaued curves typically represent lower death rates in middle age and few deaths due to illness or pathogens. The history of mouse longevity has changed dramatically with the introduction of barrier colony maintenance. In the past mouse longevity resembled current primate survivorship with linear decline in middle age. Alterations of environmental control and medical intervention have resulted in increased survivability and plateaued mid-life survivorship [25, 26]. Barrier maintenance of animal colonies typically includes controlled entry, strict quarantine procedures, disinfection protocols for all items into the animal space, controlled air flow, pure dietary sources and autoclaved water. Rodent barrier colonies implement these strategies at varying degrees depending on strain and risk of contamination from other sources [9].

Given the need for a short-lived nonhuman primate as a model of aging, the reported increased longevity from the closed Japanese colony, and the shifts in rodent survival under barrier conditions, we developed a barrier maintained specific pathogen free (SPF) marmoset colony. We hypothesized that maintaining marmosets in barrier conditions would increase marmoset longevity, alter the survivorship curves for marmosets and improve marmoset health measures. This paper describes the development of that colony, and compares health and lifespan outcomes of the SPF colony with the conventionally housed colony from which it was derived.

## RESULTS

### Establishment of SPF marmosets

Marmosets (28 males, 31 females) from Southwest National Primate Research Center (SNPRC) were screened for pathogens to identify potential founders for the SPF colony. The presence of viremia was analyzed for GB virus-A (GBV) and Callitrichine Herpes virus-3 (CHV) in 50 selected individuals. Due to the characteristics of the two viruses processing of blood samples differed prior to assay. CHV is a DNA virus related to the Epstein Barr Virus, a herpes virus. The virus is primarily present in B lymphocytes, so DNA was purified from a total blood cell pellet for PCR. GBV-A is an RNA virus distantly related to hepatitis C virus, a flavivirus, and was detected in serum derived RNA by RT-PCR. The overall prevalence for males and females was approximately the same for both viruses. The small sample size did not allow an estimation of whether the infection rate changed with age. The prevalence for CHV in a group of 50 animals ranging from 1-4 years of age was 60% with 20 negative animals (Table 1A). The same group had a prevalence of 36% for GBV-A with 32 negative animals. Repeat screening of 12 animals resulted in two discordant values for CHV. One detected the conversion of the animal to positive over a 19-month period and continued confirmation of that positive result on subsequent bleeds. Within the group of 50 animals screened, 16 animals were double negative and suitable for selection for the barrier colony. Animals were also screened for the presence of fecal pathogens including G*iardia*, *Cryptosporidium* and *Clostridium perfringens* and the frequency of positive screens are shown in Table 1B. Six females and six males were determined to be negative for all viruses and fecal pathogens of interest and were moved to the Texas Research Park to found the SPF barrier colony. An assessment for viral presence 12 months after the founding of the barrier colony revealed one male to be positive for CHV and he was moved back to SNPRC. No other animals were noted to be positive for these viruses of interest at that time, or when screened 6 years after founding the colony. Animals from the barrier colony were screened for fecal pathogens annually in years three, four and five after forming the barrier colony; the rates of positive screens are in Table 1B. Age and sex matched animals from the conventional colony were screened for fecal pathogens as well and the rates of positive screens are also in Table 1B. The prevalence of *Cryptosporidium* and *Clostridium perfringens* were very low in both the conventional and the barrier colony. *Giardia* was more prevalent than the other fecal pathogens, though prevalence of *Giardia* remained significantly lower in the barrier than in the conventional colony (F (1,8) =5.921, p=0.041).

**Table 1A:** Marmosets were screened for Callitrichine Herpes virus-3 (CHV) and GB virus-A (GBV) prior to entry into SPF colony. Number of animals positive/number of animals screened for each virus at each age screened

CHV						
Age	Females		Males		Totals	
	n	%	n	%	n	%
1	9/16	56.3	6/10	60	15/26	57.7
2	4/5	80	6/8	75	10/13	76.9
3-4	3/6	50	2/5	40	5/11	45.5
Total	16/27	59.3	14/23	60.9	30/50	60
						

GBV						
Age	Females		Males		Totals	
	n	%	n	%	n	%
1	6/16	37.5	6/10	60	12/26	46.2
2	1/5	20	1/8	12.5	2/13	15.4
3-4	2/6	33.3	2/5	40	4/11	36.4
Total	9/27	33.3	9/23	39.1	18/50	36

**Table 1B:** Number of marmosets found to be positive for pathogens during the screening process for the formation of the SPF colony, during the barrier maintenance screening, and from the conventional colony as comparison

	Sex	Number	*Giardia*	*Cryptosporidium*	*Clostridium perfringens*
**Initial Screen**	Female	15	4	1	2
	Male	18	5	1	0
**Barrier**	Female	28	2	0	0
**Maintenance**	Male	19	2	0	0
**Conventional**	Female	16	5	0	1
**Comparison**	Male	12	2	0	4

### Colony growth

The colony began with a single breeding pair that was imported in July 2007, followed by three pairs in July 2008 and four more individuals between August 2009 and April 2010. These founding six breeding pairs produced 36 litters. Breeding pairs of unrelated individuals were formed when the animals were at least two years old and between December 2007 and August 2016 the SPF barrier colony produced 98 litters consisting of 275 infants from 27 breeding females and 23 breeding males. The litters in the colony consisted of four aborted pregnancies of unknown litter size, three singletons, 20 twin, 53 triplet, 17 quadruplet and one quintuplet litter. The reproductive outcomes are summarized in Table 2. Overall the reproduction in the barrier colony was comparable to that reported for other colonies including the conventionally housed SNPRC colony (Table 2). On average the barrier colony produced two surviving infants per female per year while the SNPRC colony produces 1.6-1.8 infants per female per year. The use of rotational hand rearing in the barrier, which consisted of the rotational removal of a single infant from the family group for daily supplementation and return to the family each day, led to higher survival rates amongst triplet litters than those litters in which it wasn't implemented. For triplet litters in which all infants were born alive, rotational rearing resulted in 83.3% of infants surviving through the first 14 days, while not implementing rotational rearing resulted in 50.6% of infants surviving. If litters that were rotationally reared were removed from the analysis 97.1% of infants that survived to 14 days in the barrier colony survive to six months, 97.1% to 12 months, and 97.1% to 18 months meaning there was no loss of infants between the ages of six months and 18 months in the SPF colony. In comparison to infants at SNPRC that were not rotationally reared, lived to 14 days, and were censored for experimental deaths, 89.2% survived to six months, 81% to 12 months, and 68.4% survived to 18 months.

**Table 2A:** Reproductive outcome for the Barshop SPF barrier colony and the conventional colony at SNPRC

Infant Outcome						
Colony	Pregnancies	# of infants	# abort	# stillbirth	# infants surviving to 14 days	% Survival to 14 days
SPF Barrier	98	275	11	28	141	51
SNPRC	236	630	27	10	252	40

**Table 2B:** Reproductive outcome for the Barshop SPF barrier colony and the conventional colony at SNPRC

Litter Size					
Colony	Single	Twin	Triplet	Quad	Quintuplet
SPF Barrier	3	20	53	17	1
SNPRC	10	78	114	28	2

### Longevity in barrier

**Figure 1:**
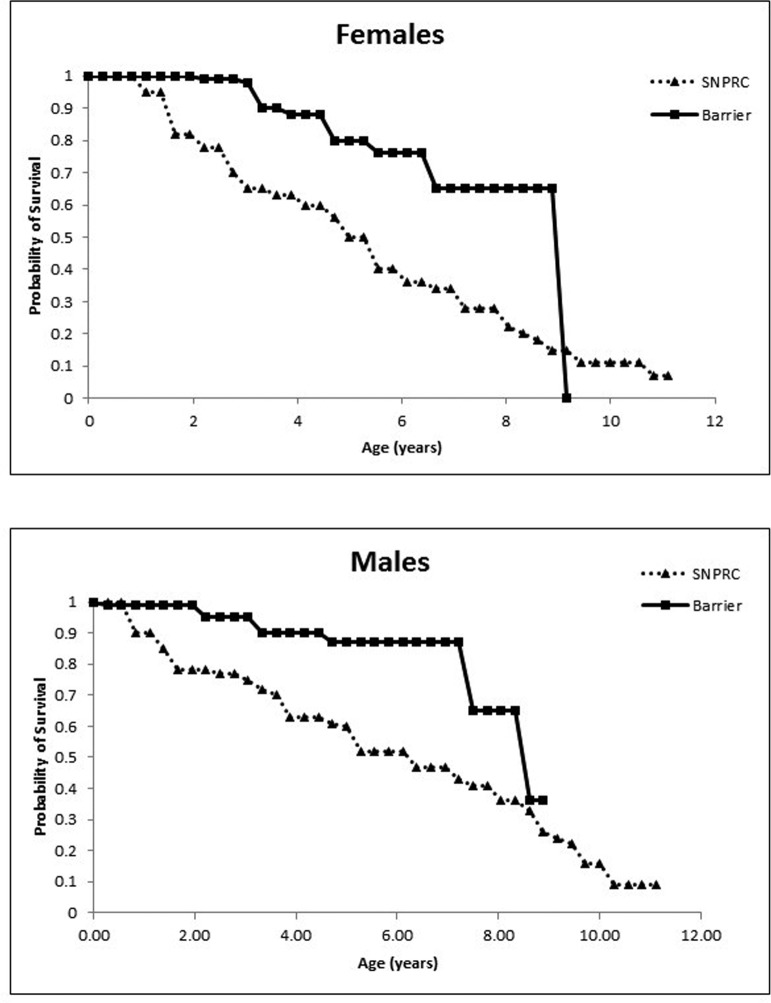
(**A**) Probability of survival for female marmosets in the Barshop SPF barrier colony and the conventionally housed SNPRC colony. (**B**) Probability of survival for males.

Maintaining marmosets in barrier conditions significantly increased the survival of animals, and adult survivorship plateaued in both males and females when compared to marmosets maintained in conventional housing protocols at SNPRC (Figure 1). We compared survival in a population of 247 barrier animals (115 females and 132 males) versus a population of 370 conventionally housed animals (173 females and 197 males) over a 10-year period. Barrier females had a median lifespan of 8.9 y (95% CI 5.9+ y, upper confidence limit could not be determined due to censoring) compared to a median lifespan of 4.86 y (95% CI 4.06 to 6.38 y) at the primate center with a hazard ratio relative to barrier females of 2.8 (p = 0.001). The female barrier survivorship curve displays a predicted precipitous decline at 9 y which we believe was due to the fact that the longest lived females died, while many middle-aged females were still alive and censored. We remain confident that a difference between the colonies exists based on the Cox proportional hazard analysis and the differences in the median lifespan and their confidence limits. Further, barrier males had a median lifespan of 8.64 y (95% CI 7.6+ y, upper limit could not be determined due to censoring) compared to the 5.08 y (95% CI 4.33 to 7.61 y) at SNPRC with a hazard ratio relative to barrier of 3.44 (p =0.001). Because the populations were tracked for no more than 10 years, there is not a valid lifespan comparison of the SPF colony data with that from the CLEA colony in Japan (Nishijima et al.,2012). However, the early to mid-adulthood survival percentages suggest that the barrier and CLEA populations are similar (Table 3).

**Table 3:** Age- and sex-specific survival percentages for barrier colony, CLEA closed colony and SNPRC conventionally-housed, open colony. (^*^ from Nishijima et al., 2012)

		Survival		
Colony	Sex	2 years	5 years	8 years
Barrier	Male	100	90	68
	Female	100	80	68
CLEA*	Male	95	92	87
	Female	92	78	62
SNPRC	Male	78	60	38
	Female	80	50	22

### Health outcome

There were significant differences in the blood cell counts of barrier and SNPRC animals, including white blood cell (F (1,42) = 14.586, p<0.001), monocyte (F (1,42) = 14.808, p<0.001) and neutrophils (F (1,42) = 17.208, p<0.001) (Table 4). Matched comparison of blood chemistry values also showed significant differences between colonies for albumin (F1,26) = 8.032, p=0.009), globulin (F1,26) = 8.019, p = 0.009), and A/G ratio (F (1,26) =11.075, p=0.003). Regardless of which colony the animal was from, age was significantly correlated with lymphocyte count (r = −0.311, p=0.04), alkaline (r= 0.551, p=0.002), globulin (r= 0.57, p=0.001), BUN (r= −0.434, p=0.17), A/G (r= −0.516, p=0.003), and BC (r= −0.425, p=0.019). There were no significant changes in blood parameters for individuals over the 18 months of sampling regardless of location. There were also no significant differences in any measured urinary parameters between the locations.

**Table 4:** Average blood chemistry values (+ standard error) for marmosets maintained in the Barshop SPF barrier colony and the conventional SNPRC marmoset colony (^*^significant at p< 0.05)

Test	SPF Barrier	SNPRC	Normal Range	UNIT
WBC*	6.06 ± 0.06	10.7 ± 0.09	1.8 - 8.1	(Thous/MM3)
BASO	0.08 ± 0.02	0.13 ± 0.03	0 - 0.1	(Thous/MM3)
EOS	0.05 ± 0.02	0.1 ± 0.03	0 - 0.2	(Thous/MM3)
MONO*	0.29 ± 0.04	0.86 ± 0.2	0 - 0.6	(Thous/MM3)
LYMPHS	3.7 ± 0.4	4.2 ± 0.4	0.7 - 5.0	(Thous/MM3)
NEUT*	2.6 ± 0.3	5.4 ± 0.7	1.5 - 8	(Thous/MM3)
ALK	138.4 ± 19.3	183.5 ± 22.2	5 - 113	U/L
ALT	13.2 ± 3.9	15.9 ± 4.6	0 - 62	U/L
ALBUMIN*	4.6 ± 0.09	4.2 ± 0.11	2.9 - 5.2	g/dL
PROTEIN	7.4 ± 0.12	7.35 ± 0.14	5.2 - 8.1	g/dL
GLOBULIN*	2.77 ± 0.08	3.15 ± 0.1	2.1 - 3.2	g/dL
BUN	27.97 ± 1.46	25.1 ± 1.7	12 - 31	mg/dL
CREATININE	0.36 ± 0.02	0.4 ± 0.02	0.3 - 0.5	mg/dL
GLUCOSE	166.9 ± 12.2	152.5 ± 14.04	92 - 244	mg/dL
A/G RATIO*	1.68 ± 0.06	1.37 ± 0.07	1.1 - 2	
B/C RATIO	80.3 ± 4.7	63.1 ± 5.4	30 - 81.2	

There were very few adult deaths during the tenure of the barrier colony. There were only 12 female deaths and 7 male deaths from the original founders and the 141 animals that survived past the age of 14 days. From these deaths there were only a few for which conclusive pathology and cause of death were determined. A male age 4.5 y died from congestive heart failure and epilepsy. A male 2 y old appears to have died due to complications associated with diabetes. A male juvenile age 6 months died from congenital pancreatic failure. Siblings age 3.5 y died unexpectedly, the male from congenital gastrointestinal defect and the female from anemia. A female 4.5 y died from postpartum complications. A female's death was associated with cardiac and renal failure at 4.3 y. For several deaths in the colony we do not have conclusive pathology or cause of death, we present here anecdotal symptomology noted around time of death. A male age 7.6 y was found with a rectal prolapse of unknown etiology immediately prior to death. A female age 9 y exhibited signs of dehydration following infant delivery shortly prior to her death. Her mate age 8.6 y was noted to have enlarged hardened kidneys during a physical prior to his death of unknown cause. One infant male age 22 days was being supplemented with formula and may have aspirated during feeding. For the other 11 deaths there were no reported clinical symptoms or signs prior to death. There is no evidence from the pathology reports that any animals in the barrier died due to inflammatory gastrointestinal or infectious disease.

By contrast, in the comparative SNPRC population there were five deaths of animals under the age of one year for which the cause of death was determined to be necrotizing colitis, two that were noted as failure to thrive infants with extensive amyloidosis, two with *Pseudomonas* septicemia, and three with diarrhea associated with colitis. Between the ages of one year and two years four animals died due to necrotizing colitis, eight deaths were colitis associated, three deaths were associated with pneumonia, one death was due to cholangitis, two deaths were a result of trauma, and two were associated with amyloidosis. For animals aged 2-5 y there was one death associated with necrotizing colitis, six due to colitis, one due to pneumonia, one due to cholangitis, five associated with amyloidosis, two individuals with lymphoma, three deaths due to nephritis, one animal died due to cardiomegaly, and one female died during delivery due to dystocia. For animals between the ages of 5-8 y there were seven deaths associated with colitis, one death due to cholangitis, one death due to lymphoma, one associated with nephritis and one female death due to postpartum complications. For animals over the age of eight there were two deaths associated with colitis, two deaths due to amyloidosis, three deaths due to nephritis, and five deaths due to cardiomyopathy.

### Microbial communities

**Figure 2:**
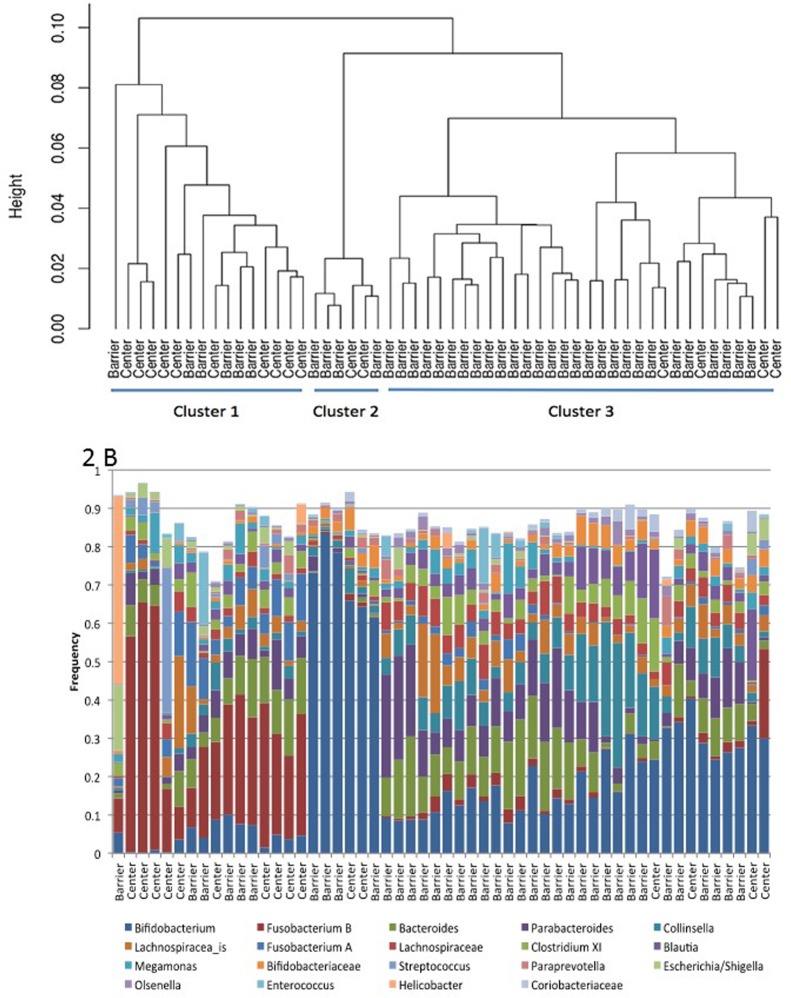
(**A**) Hierarchical clustering of marmoset gastrointestinal microbiomes from Barshop SPF barrier colony (Barrier) and the conventionally housed SNPRC colony (Center). (**B**) Frequency of taxa used in the cluster analysis, ordered by position in (**A**).

The lack of gastrointestinal problems in the SPF barrier colony and the litany of gastrointestinal problems observed in the SNPRC animals might be linked to differences in the composition and function of microbial communities in their gastrointestinal tracts. Sequences of the 16S ribosomal RNA gene were used to characterize microbial community composition for each of 38 SPF marmosets and 16 SNPRC marmosets. A subset of 19 microbial taxa were used in the comparison because these taxa occurred in at least one sample at a frequency of 5%. A hierarchical cluster analysis of the microbial communities based upon the frequencies of these taxa was performed (Figure 2). This analysis showed that the gastrointestinal microbiomes of barrier and SNPRC animals differed in terms of the kinds and abundances of various bacterial taxa (Figure 2B). There were three major clusters that grouped together microbiomes with similar bacterial compositions. The frequencies of the clusters in the two colonies were significantly different (Table 5; χ^2^= 12.8, simulations=5,000, p-value< 0.002) [27].

**Table 5:** Frequency of microbiome clusters in the Barshop SPF barrier colony and the conventional SNPRC marmoset colony (significant at p< 0.002)

Colony (n)	Cluster 1	Cluster 2	Cluster 3
Barrier (38)	16%	10%	74%
Conventional (16)	62%	13%	25%

The frequencies of *Bifidobacterium* and *Fusobacterium* B (a group that is mischaracterized as *Clostridium* XIX) largely determined membership within a given cluster, and they were negatively correlated with each other (Figure 2). Thus, communities in Cluster 1, which was the predominant type in the SNPRC colony, had comparatively high frequencies of *Fusobacterium* B (>6%, median=27%) and low frequencies of *Bifidobacterium* (<8%, median=4%). In contrast, communities in Cluster 3, which was predominant in the barrier colony, had higher frequencies of *Bifidobacterium* (median=17%) and lower frequencies of *Fusobacterium* B (median=1%) with only a single exception, which had high frequencies of both. Communities of Cluster 2 had the highest frequencies of *Bifidobacterium*, >50% (median=70%) and the lowest frequencies of *Fusobacterium* B (median=0.0%). Animals with these communities were infrequent in both colonies.

## DISCUSSION

One of the techniques in animal husbandry that has helped to reduce deaths due to infectious disease, and to increase the likelihood that deaths are due to age rather than other environmental factors, is the development of specific pathogen free colonies with implemented barrier conditions. Rodent colonies for biomedical research began implementing procedures for deriving specific pathogen free lineages in the 1980's and husbandry practices altered dramatically to include barrier protocols [25, 26, 28]. The implementation of barrier protocols has resulted in major changes to the reported maximum lifespan for many mouse lineages, for example 129/J mice were reported to have an increase in lifespan of 22% from 648 days in 1966 to 791 days in 2009 [29]. While there may be a variety of causes for this increased lifespan, most researchers and colony managers agree that the implementation of HEPA filtered air systems, controlled diets, and sealed rack units have resulted in decreased infectious disease spread and decreased stress for the animals. The implementation of these husbandry practices was not just beneficial to animal welfare, health and aging, it has resulted in improved health for the caregivers, and increased quality and repeatability of research between research sites [25].

Establishment of nonhuman primate SPF colonies have been largely driven by the needs of specific research agendas and not to support the development of aging studies. The most well-established SPF nonhuman primate is the rhesus macaque which has been cleared of tuberculosis, simian retrovirus (SRV), simian immunodeficiency virus (SIV), and Herpes B virus [30]. As opposed to our efforts, the main driving force for the establishment of these SPF rhesus macaque colonies has been to support SIV/AIDS research [30], as well as clearing the species of a pathogen that is potentially fatal to humans. A baboon colony that is SPF for five herpesviruses, four retroviruses, simian virus 40, measles and monkey pox was established at the Oklahoma Health Science Center [31], primarily to support the use of baboons in transplantation studies requiring immunosuppression. The Barshop marmoset colony was the first new world monkey self-propagating SPF colony to be developed. Marmosets were selected as a species in which to develop an SPF barrier colony for aging research because their lifespan, size and social characteristics make them ideal for biomedical research and specifically aging research. Marmosets do not pose a zoonotic risk for human caretakers and researchers since they are not carriers of the herpes B virus that macaques carry [32].

The choice of pathogens to screen for and remove in the development of the marmoset SPF colony was complicated by our limited understanding of the relationship of viral infections to disease in this species. One of the viruses (CHV) has been proposed to be a possible cause of lymphomas, however others dispute this claim [33, 34]. The screen for viral, bacterial and parasitic pathogen presence revealed few animals that were free from all identified agents. Interestingly the SPF colony was able to be derived without the need for removing infants from the mother shortly after delivery and nursery rearing with other infants, or the more stringent cesarean derivation that has been implemented in a number of SPF colonies [30, 31, 35]. Adult marmosets were identified that were negative for both viruses and the identified bacterial and parasitic pathogens of interest. With the exception of one male, all animals remained negative for the viruses, and few were found to be positive for *Giardia* during the years of follow-up. This is quite different from the viruses of interest in Old World monkey SPF colonies such as herpes B, which sporadically break through, appearing in previously sera-negative animals either due to latent tendencies or *in utero* transmission. Thus, the marmoset SPF colony could be derived without the use of infant isolation from the family group to maintain viral negative animals.

Preventing bacterial and parasitic pathogens was not as easy, with *Giardia* being prevalent in the conventional colony and appearing sporadically in the SPF colony. While *Giardia* can be treated with Tinidazole, the rate of false positives in the assays and the inability to detect it during the latency period made it more difficult to remove from the SPF colony. However, *Giardia* was noted to occur at significantly lower rates and did not spread through the SPF colony. Animals in the SPF colony that had a positive *Giardia* result were tested regularly as follow-up and none had a repeat positive result, whereas animals in the SNPRC colony did repeatedly test positive and were treated with Tinidazole. Further, we did not have deaths in the SPF colony that were directly associated with gastrointestinal disease which has been associated with intestinal infections including *Giardia* [36].

The Barshop marmoset colony quickly established as a self-propagating SPF breeding colony in which infants were successfully raised by their SPF parents. In fact, the production and success of the SPF colony was significantly better than the conventional colony. While rotational hand-rearing and investment by the marmoset staff in early infants did increase the rate of success for triplet litters, the overall success of the colony was better for all litter types. The elimination of specific pathogens and maintenance in strict barrier conditions increased infant survivorship and decreased juvenile and early adult deaths. Of particular importance for establishing aging research colonies, marmosets can be bred and continually housed socially under barrier conditions – something that is decidedly more difficult to accomplish with large-bodied Old World monkeys.

There were differences in some blood chemistry values between the SPF and conventional colonies. Most of the blood and urinary values fell well within normal range for marmosets. The SPF colony did exhibit significantly decreased white blood cell, monocyte and neutrophil count when compared to the conventional colony. While typically within the normal range, the conventional colony had individuals with values at the top of the range. These results suggest that on average the marmosets in the SPF colony had lower antigen exposure rates than the conventional colony.

The Barshop colony had significantly increased early and mid-adult age-specific survivorship when compared to most other marmoset colonies. The results were similar to those of the colony that reported the highest average median and maximum life span [37]. One interesting difference in the SPF barrier colony from both the SNPRC conventional colony and the CLEA colony is the lack of a sex difference in survivorship. Nishijima et al. (2012) report significantly lower age-specific survival in marmoset females than in males, a finding that we have also reported previously for the SNPRC marmoset colony [38]. The reasons for this difference are unclear.

The New England Primate Center previously reported causes of death within their marmoset colony between 2004 and 2009 as primarily inflammatory bowel disease, conspecific injury, and infectious disease of the gut and kidney [21]. The primary causes of death for marmosets under the age of 6 at SNPRC between 2002 and 2011 were irritable bowel disease and colitis [20]. These causes of death for animals under the age of 6 were not seen in the Barshop SPF colony. Coincident with these differences in causes of death is the striking difference in the gastrointestinal tract microbiomes of the SPF and SNPRC colonies. Whether these differences are due to the selection of founding animals with specific microbiomes, or to changes that occurred after the colony was established is unknown. The differences may also reflect differences in the environmental conditions and/or the exclusion of specific organisms. It would certainly be interesting to know whether the improved health in the barrier colony, and the lack of deaths due to intestinal diseases are related to the differences in microbial community composition.

The plateau effect for adult longevity depicted by the barrier colony is presumably due to the decreased exposure to infectious agents and pathogens rather than genetic effects on longevity since the barrier colony was founded from the SNPRC conventional colony. All of the health and colony indicators suggest that maintenance in a barrier reduced early mortality, increased colony rate of growth, and supported significantly healthier marmosets. The ability to detect differences in lifespan and mortality rates in association with colony management and husbandry techniques may indeed be valuable for future aging research.

A future question to address is the extent to which barrier procedures, such as autoclaved water, irradiated food, high air exchanges, and enhanced personal protective equipment contributes to these improved outcomes, as opposed to the simple fact of having a closed colony. We have previously reported that we have observed improvements in early-adult survivorship when the SNPRC colony was retained as closed (2001-2006) and those improvements disappeared when the colony was opened to importation of new animals to support colony growth [21]. In addition, the CLEA colony has been closed to new animals for over three decades but does not report using barrier techniques for maintenance. It is possible that simply preventing the influx of pathogens from outside sources is enough to increase longevity and health-span in marmosets and that full barrier techniques are not necessary. However, almost all rodent models of aging and health-span are maintained under barrier protocols, and the ability to translate results from rodent experimentation to primate models require barrier protocols to be designed and evaluated for primates. While there is certainly room for further enhancement and testing of barrier techniques for use with marmosets, the Barshop SPF barrier colony firmly supports that marmosets can be bred and maintained in an environment ideal for aging research, and the health and longevity of these animals is improved over conventionally housed animals.

## CONCLUSIONS

Marmosets offer a unique model for biomedical research and for aging studies in particular. The shorter lifespan, small size, and ease of care make them ideal as a nonhuman primate model. The ability to successfully create a specific pathogen free colony of breeding marmosets offers the opportunity to broaden their use by researchers to investigate causes of age related decline and biological age related disease.

## METHODS

### Specific pathogen free marmosets

The SPF barrier marmoset colony was begun at the Barshop Institute for Longevity and Aging Studies, University of Texas Health Science Center at San Antonio in 2006. In order to populate this colony, animals from the Southwest National Primate Research Center were screened for several identified agents associated with pathogenic outcomes. Blood from animals of interest was screened for the presence of the viruses GB virus-A (GBV) and Callitrichine Herpes virus-3 (CHV) by the Lanford viral core at the Texas Biomedical Research Institute [39, 40]. Marmoset blood was obtained with EDTA anticoagulant. Plasma was removed after centrifugation. The cell pellet was suspended with PBS to 1 ml. DNA was purified from the 200 μl of the cell suspension using a Qiagen mini-DNA column purification kit. DNA was eluted in a volume of 25 μl of water and the concentration was determined by using a Nanodrop ND-1000. TaqMan real time PCR amplification for CHV DNA was performed with 200 ng of cell DNA using an ABI 7500 TaqMan machine with the ABI Fast Advance PCR solution. The TaqMan primers and probe were designed against the major internal repeat of CHV. The primers and probe were: forward primer (TGGGCCTAGTCTCCCCATAGA), reverse primer (GTGAGGGAGTCCATAAGGAAACTTT), and a Fam-Tamra labeled probe (CGCCTGTATGTCTTACTGGGACCCCTG). The RT-PCR assay for GBV-A RNA was a gel based assay. Multiple attempts to develop a quantitative TaqMan RT-PCR were not successful. The sequence of GBV-A was not available from multiple isolates. The target region selected for this assay was based on available sequences and was variable (Buhk). Sequence of the target region from animals within the SNPRC colony, confirmed the sequence variation confounding the ability to develop a probe for the detection of all isolates. The RT-PCR used for this project employed a single round of 40 cycles of amplification followed by detection of the product by agarose gel electrophoresis and ethidium bromide staining. RNA was purified from 50 μl of plasma using RNA-Bee following the manufacturer's protocol. The final RNA pellet was suspended in 50 μl of water and 10 μl was amplified using the Invitrogen RT-PCR Superscript Kit for Long Templates. The primers detected a 400 nucleotide region in the 5′untranslated region (UTR) of marmoset GBV-A. The primers were: forward primer (AGGGTTCGTAGGTGGTAAATCCC) and reverse primer (TGCCACCAGGGGTCACCCGAAG).

Fecal samples from candidate animals were evaluated for the presence of *Giardia* spp., *Cryptosporidium* spp., *Clostridium perfringens*, helminths, parasitic ova, enteropathic *E. coli*, *Campylobacter* spp., and *Trichimonas* spp. through IDEXX commercial veterinary screening services. In initial screenings of the SNPRC colony no marmoset tested positive for the presence of *Campylobacter* spp. or *Trichimonas* spp. and these assays were dropped from further assessments. Screening for all other pathogens was done prior to colony formation and then periodically after the formation of the SPF colony. The first SPF male-female pair of marmosets was moved to the barrier facility at the Barshop Institute July 2007. The initial formation of the colony consisted of a total of 12 founder animals, with animals entering between July 2007 and April 2010. In order to assess the maintenance of the colony as specific pathogen free, viral screening occurred for all animals six months after entry to the barrier and sentry animals (one from each family group) were six years after formation of the colony. Fecal collections to assess the presence of *Giardia*, *Cryptosporidium*, and *Clostridium perfringens* were done annually following the formation of the colony.

### Barrier maintenance

**Figure 3:**
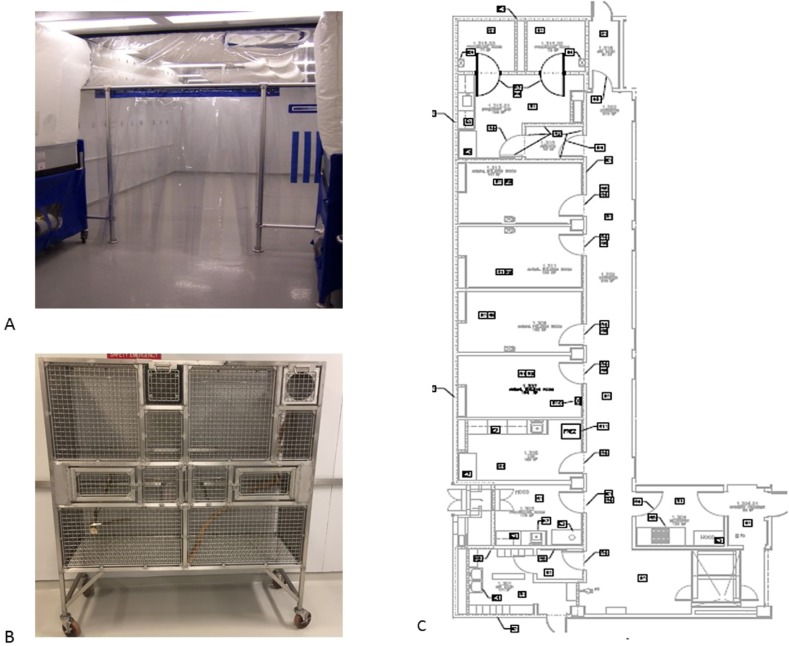
(**A**) Barrier space at the Barshop Institute with bioBubble airshower units. (**B**) Stainless steel marmoset breeding cage (LG). (**C**) Layout of converted BSL3 Barrier space for marmoset colony.

The first barrier rooms of the colony were two 10′x 29′ rooms within the animal care facility at the Barshop Institute located at the UT Health San Antonio Texas Research Park. Rooms were equipped with bioBubble units (Fort Collins CO, www.biobubble.com) consisting of two HEPA filtered airshower power units providing 200-300 air changes per hour in the 10′ × 18′ animal vivarium space (Figure 3A). The front entry of the room contained clean PPE storage for entry into the screened vivarium. Each room maintained a positive air pressure to the outside hall. All items within the bioBubble and entering the bioBubble area were considered clean and rigorous SOP's to ensure cleanliness were used. The vivarium housed six family units consisting of at most two adults, four independent offspring and two-three dependent offspring in marmoset breeding cages measuring (1.3 × 1.5 × 0.6m) made of stainless steel specifically designed for this colony (LGL) (Figure 3B). All items entering the barrier were sanitized prior to entry or autoclaved. Water bottles were autoclaved before use, and all water used in the room was autoclaved. The paper placed under the cage to capture waste was DACB neomycin coated cage liner (Shepherd Specialty Paper, Watertown, TN) to decrease the potential for aerosolized urinary product and contamination between cages. Personal protective equipment for the facility included standard shoe covers, hair bonnet, face mask and gloves as well as a disposable outer gown. Staff was required to enter the barrier rooms prior to work with any other animals or entry into other animal facilities. Clean scrubs were required to enter the barrier spaces, and if a staff member had already entered another animal room they were required to shower before entering the marmoset barrier.

SPF marmosets in the barrier were fed a purified diet to prevent food borne pathogens from entering the colony. The Harlan Teklad purified marmoset diet (TD99468) used by SNPRC [41] was modified to be irradiated at the production factory and double bagged in 1kg portions. This purified diet was prepared under a hood using gelatin as the setting agent and autoclaved water. The food containers were sealed under the hood and transported to a refrigerator in the animal room. Due to the unusual nature of the facility food enrichment was restricted within the barrier. An irradiated primate enrichment mix consisting of nuts, seeds, and dried fruit from Harlan Teklad was given daily. In cases of ill animals, or animals that were deemed to need supplements, items were chosen that could be purchased in sealed single use containers such as baby food or high calorie nutrition drinks, and unused portions were disposed of daily.

In May 2013 the SPF marmoset colony was moved to a new Barrier space at the Barshop Institute (Figure 3C). This new barrier space was 2850 ft^2^ decommissioned and reconfigured BSL3 space in the animal vivarium. This new barrier facility consisted of a pass-through double door entry, a food preparation room, laboratory procedure room, four animal holding rooms (∼180ft^2^ each), a surgical suite, and 2 autoclave entry areas. The isolated air filtration system in this space was modified such that instead of HEPA filtration preventing contaminants from leaving the space the filtration prevented contaminants from entering the space. The entire space once entering through the pass through double door entry was considered clean barrier space. The air exchange in this facility was also maintained at the higher rate of 200 changes per hour. Humidifiers (Humidifirst) were added to each animal holding room that use double filtered RO water to maintain room humidity between 35 and 40%. Barrier protocols were maintained similarly to the bioBubble space and all other general maintenance and husbandry protocols were adopted from SNPRC's marmoset colony [42]. The research adhered to ethical guidelines for primatological research as outlined by the American Society of Primatologists and the Institutional Animal Care and Use Committee at the University of Texas Health Science Center San Antonio and SNPRC.

### Health monitoring

To compare the health outcomes for SPF marmosets maintained in a barrier to conventionally housed marmosets we evaluated blood CBC and blood chemistry of all animals in the barrier to age and sex matched animals maintained in the SNPRC marmoset breeding colony. All of the blood counts were done in the Tardif lab by a single technician, and the blood chemistry samples were sent to IDEXX for evaluation.

Additionally, urine was collected for these age- and sex-matched individuals and basic urinary values were assessed using a strip test to establish values of protein, glucose, ketone, specific gravity and presence of blood in the urine. Results from the two colonies were compared statistically using SPSS 23.0 repeated measures ANOVA.

To examine longevity in the SPF colony, age at death for individuals in the barrier were compared to the SNPRC colony records for age of death during the same date frame. We compared survival in a population of 247 barrier animals (115 females and 132 males) versus a population of 370 conventionally housed animals (173 females and 197 males) over a 10-year period. The final date of comparison was August 2016 and animals were censored from the data if they were still alive at the end of date frame, if they had been transferred to another primate facility, or if death was associated with an experimental protocol. For marmosets, the first two weeks of life present a high risk of neonatal death and are typically removed from longevity analyses, as we did here. Survivorship curves were estimated using Kaplan-Meier estimation, Cox proportional hazard model and Fisher's exact test with males and females evaluated separately.

### Microbial community analysis

To evaluate the microbial diversity of the marmoset gastrointestinal tract, 38 marmosets living in the barrier colony and 16 marmosets from the conventionally housed SNPRC colony were sampled between October, 2011 and April, 2012. A fecal sample was taken from each marmoset by inserting a mini e-swab (Copan Diagnostics) into the rectum. E-swabs were stored in Amies transport medium and immediately frozen at −80°C. Samples were mailed to the University of Idaho on dry ice and stored at −80°C. Using methodology that is standard at the University of Idaho [43], DNA was extracted from the fecal samples, and the V1-V3 region of the 16S ribosomal RNA gene (universal 16S rRNA primers 27F and 534R) was amplified and then sequenced by an Illumina MiSeq. Sequence reads were processed for quality and assigned to best taxonomic level by the IBEST Genomics Resources Core (http://www.ibest.uidaho.edu/cores/genomic-resources-core/). The depth of coverage for each community was sufficient to detect taxa that constituted ≈0.01% of a community. Taxa that occurred at low frequency were removed from further consideration (ie: if a taxon had no single sample with a frequency greater than 5%).

The R statistical package, vegan, was used to calculate alternative Gower distances among samples based upon taxon frequencies, and these distances were clustered by a hierarchical algorithm, hclust, using complete linkage. The number of clusters was determined by eye and confirmed using silhouette plots. The number of samples from barrier and conventional marmosets were enumerated for each cluster, and Fisher's exact test using simulated p-values was used to test for random associations between cluster and colony type. A stepwise discriminant analysis was used to determine whether specific bacteria contribute to the placement of samples within clusters.
